# Difficult Airway Management of a Patient With Sturge-Weber Syndrome: A Case Report

**DOI:** 10.7759/cureus.113929

**Published:** 2026-08-04

**Authors:** Haruka Hoshi, Shigekazu Sugino, Kotoe Kamata, Masanori Yamauchi

**Affiliations:** 1 Department of Anesthesiology and Perioperative Medicine, Tohoku University School of Medicine, Sendai, JPN

**Keywords:** airway management, difficult airway management, intubation difficulty, sturge-weber syndrome, video-laryngoscope

## Abstract

Sturge-Weber syndrome is a rare neurocutaneous disorder characterized by vascular malformations of the brain, eyes, and face, which can lead to difficult airway management. A 57-year-old man with Sturge-Weber syndrome presented with an oral hemangioma. Preoperative assessment, including computed tomography, raised concerns about anticipated difficult mask ventilation and tracheal intubation. Based on these findings, awake intubation was planned. After sedation and topical anesthesia, a video laryngoscope was used to visualize the glottis, and the trachea was successfully intubated. General anesthesia was subsequently induced and maintained, and the surgery proceeded without complications. The patient was extubated after confirmation of the absence of oral bleeding or other airway abnormalities. Careful preoperative airway assessment is important in patients with Sturge-Weber syndrome. Awake intubation using a video laryngoscope may be an appropriate option for adult patients with anticipated difficult mask ventilation and tracheal intubation secondary to hemangiomas.

## Introduction

Sturge-Weber syndrome is a rare neurocutaneous syndrome affecting only 1 in 50,000 people, characterized by vascular malformations or hemangiomas of the brain, eye, and face [1]. Brain and eye hemangiomas can cause seizures, intellectual disabilities, neurological deficits, glaucoma, and retinal detachment [1]. Facial vascular malformations and hemangiomas affect the neck, oral cavity, and upper airway structures, such as the nose, palate, gingiva, tongue, larynx, and trachea [2]. These abnormalities render mask ventilation or tracheal intubation difficult [3,4]. Moreover, hemangiomas are prone to bleeding even with mild physical stimulation. Bleeding from upper airway angiomas raises concerns about difficult airway management [5]. Previous　case reports have indicated anticipated difficult airway in patients with Sturge-Weber syndrome [1,5-8]. Here, we report the successful management of an anticipated difficult airway in a middle-aged man with Sturge-Weber syndrome. Most reports of anesthetic management for this syndrome involve children under 10 years old; however, this report concerns an adult patient in whom awake intubation, a technique difficult to perform in pediatric patients, was used for management, and few studies have focused on this point. This case report was prepared following the Anaesthesia Case Report (ACRE) checklist: modified CARE guidelines [9].

This work was presented in part at the 15th Annual Meeting of the Japanese Society of Anesthesiologists, Hokkaido-Tohoku Branch, on September 13, 2025, in Sendai, Japan.

## Case presentation

**Figure 1:**
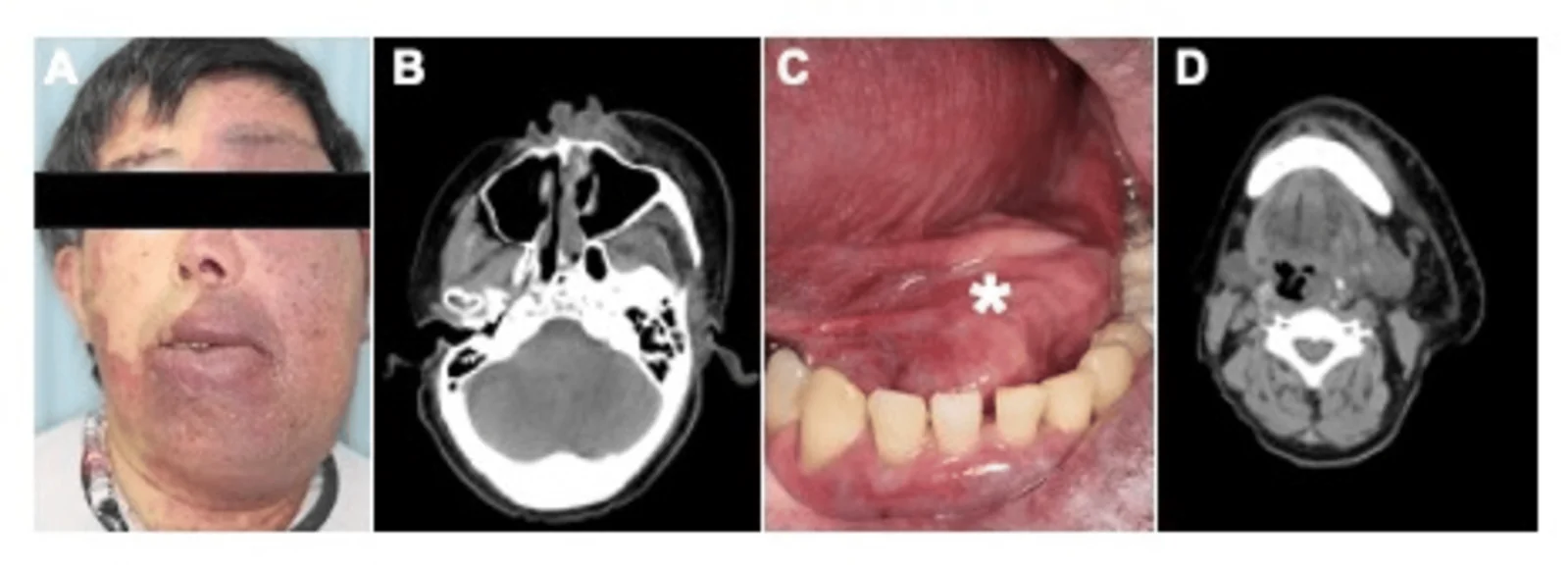
Preanesthetic evaluation of the patient. (A) Port-wine stain on the face and neck. Significant enlargement on the left side due to hemangioma. (B) CT image at the cranial base level. Hemangioma obstruction in the left nasal cavity (arrowheads). (C) Hemangioma on the left side of the tongue at the floor of the mouth (asterisk). (D) CT image at the hyoid bone level. Oral stenosis due to hemangioma swelling.

A 57-year-old man (Asian, 55 kg, 159 cm) with Sturge-Weber syndrome presented for resection of a hemangioma in the oral cavity. He had a history of mild intellectual disability, epilepsy, glaucoma, and hearing impairment. He underwent surgery to resect a hemangioma 26 years ago, but the details were unknown. He had vascular malformations with hypertrophy on his face and neck (i.e., port-wine stain), which were enlarged and had poor mobility (Figure 1A). An anesthesiologist (KK) evaluated the patient in a preoperative optimization clinic three weeks before surgery. 

Physical examination revealed reduced facial mechanical flexibility and limited tongue movements. The Mallampati classification was class II [10]. The interincisor distance was 4 finger breadths. The hyoid-mental distance was 3.5 finger breadths. The thyromental distance was 2.5 finger breadths. Poor cervical spine mobility, receding chin, prominent incisors, and short muscular neck were absent in the patient. Computed tomography (CT) showed the progression of hemangiomas involving the upper and lower gingiva, masseter, and left temporalis muscles, with associated soft-tissue swelling and nasal cavity obstruction (Figure 1B). Oral stenosis was caused by a hemangioma on the left side of the tongue at the floor of the mouth (Figure 1C, D). No progression of the hemangioma into the trachea or bronchi was noted. There were significant concerns about difficult mask ventilation during anesthesia induction and tracheal intubation because of the risk of bleeding from intraoral hemangiomas during laryngoscope manipulation.

**Figure 2:**
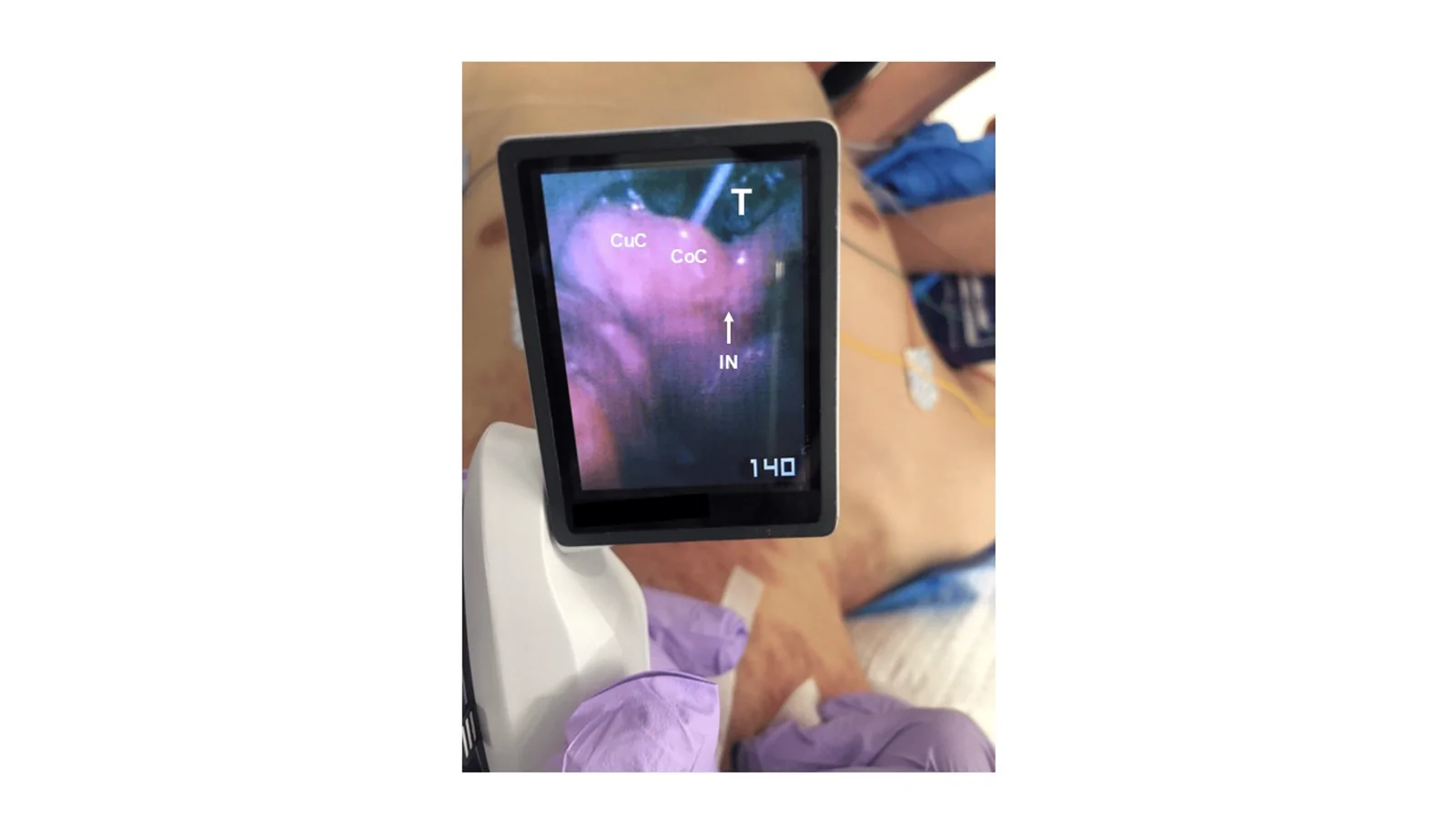
Video laryngoscope view during tracheal intubation. T: intubated endotracheal tube; CuC: left cuneiform cartilage; CoC: left corniculate cartilage; In: interarytenoid notch.

Two anesthesiologists (HH and SS) planned awake tracheal intubation to induce anesthesia. The patient’s blood pressure upon entering the operating room was 135/85 mmHg, and his heart rate was 55 bpm. First, we showed the patient a video laryngoscope (McGrath MAC with Macintosh blade, size 4) and carefully explained and demonstrated the steps of awake intubation in detail to alleviate his anxiety. After preoxygenation with 100% oxygen at 6 L/min, 1 mg midazolam and 150 μg fentanyl were administered for sedation to minimize discomfort during awake laryngoscopy. Topical anesthesia of the upper airway was achieved using 8% lidocaine spray. Topical lidocaine was first applied to the base of the tongue in four sprays. Subsequently, the oropharynx, hypopharynx, and laryngeal structures were sprayed (a total of 104 mg of lidocaine). The video laryngoscope was gently inserted into the oral cavity of the patient. Laryngoscopy revealed a Cormack-Lehane grade II view [11], with the glottis clearly visible on the monitor (Figure 2). An 8.0-mm cuffed endotracheal tube was advanced during inspiration and secured at 23 cm, fixed at the right corner of the mouth. The patient was cooperative during awake intubation. Immediately after intubation, blood pressure was 130/82 mmHg, and the heart rate was 65 bpm. Hypoxemia or intraoral bleeding occurred during anesthesia induction.

Arterial and peripheral venous catheters were inserted, and quantitative neuromuscular monitoring was used. General anesthesia was maintained with a propofol infusion at 3.6 mg/kg/h, a remifentanil infusion at 0.24 μg/kg/min, and 85 mg of rocuronium. Plastic surgeons resected hemangiomas of the buccal mucosa, tongue apex, and left side of the tongue at the floor of the mouth. The operative duration was 74 min.

At the end of the surgery, the anesthesiologists evaluated the oral cavity using a laryngoscope and observed adequate tongue mobility and the absence of bleeding. Neuromuscular blockade was reversed with 150 mg of sugammadex. The trachea was extubated after full recovery from neuromuscular blockade (train-of-four ratio 1.0), return of spontaneous breathing with adequate oxygenation and ventilation, and sufficient awakening to follow simple verbal commands. After extubation, the Glasgow Coma Scale score was E4V5M6. The patient was admitted to the intensive care unit for postoperative management and transferred to the ward on postoperative day 1. The remainder of his hospital stay was uneventful, and he was discharged on hospital day 32 in a stable condition.

## Discussion

We successfully managed anesthesia and a difficult airway in a patient with Sturge-Weber syndrome. The major concern in the anesthetic management of patients with Sturge-Weber syndrome is difficult airway management. Because Sturge-Weber syndrome is a rare disease, there are few published reports on anesthesia management in patients with this syndrome [1,12]. Anesthesiologists must carefully assess and plan the anesthesia for these patients [13].

In this case, the patient’s anatomical features indicated that mask ventilation would potentially be difficult (Figure 1). The Japanese Society of Anesthesiologists Airway Management Guideline recommends that awake intubation should be considered in cases of preoperatively anticipated difficult mask ventilation [14,15]. The American Society of Anesthesiologists’ difficult airway algorithm also recommends awake intubation in such cases [16]. Therefore, we planned to perform awake intubation.

Alternatively, the option of introducing general anesthesia could be considered to minimize patient discomfort. In this case, mask ventilation might have been achievable after rapid induction and neuromuscular blockade, as the nasal cavity was not completely obstructed, and insertion of an oral airway may have facilitated ventilation. However, inserting an oral airway carries the risk of bleeding from intraoral hemangiomas. Further bleeding could impair visualization and necessitate repeated intubation attempts. Repeated laryngoscopy and intubation attempts increase the risk of a “cannot intubate, cannot ventilate” (CICV) situation [17,18]. One advantage of awake intubation is improved safety, achieved by preserving spontaneous respiration and airway protective reflexes, thereby maintaining airway patency and reducing the risk of pulmonary aspiration of blood [14]. Nevertheless, if the patient became uncooperative, developed agitation, or became oversedated, we planned to induce general anesthesia. After loss of consciousness, an oral airway was inserted, and mask ventilation was performed manually. If ventilation was inadequate, a supraglottic airway was gently inserted. However, because surgery could not be performed using the supraglottic airway, we explained in advance that we might discontinue the surgery and awaken the patient from general anesthesia. Based on these considerations, awake intubation was considered the most appropriate airway management strategy for this patient.

The pathophysiology of Sturge-Weber syndrome involves hemangiomatosis [1]. Hemangiomas are prone to bleeding because of abnormal vascular proliferation. Forceful airway manipulation or surgical procedures may therefore increase the risk of bleeding or hematoma formation. Because gentle laryngoscopy and minimal mechanical stimulation are essential, a video laryngoscope was used in this case. Video laryngoscope-guided intubation improves airway visualization and may help reduce the risk of trauma to airway hemangiomas. Postoperative bleeding was also a concern; therefore, emergence from anesthesia was carefully managed, and awake extubation was performed only after confirming the absence of abnormalities in the deep oral cavity using a video laryngoscope.

This case highlights the importance of careful preoperative physical examination and airway imaging in the anesthetic management of patients with Sturge-Weber syndrome. Although many patients present with facial port-wine stains, which may suggest difficult mask ventilation, imaging assessment of facial and intraoral hemangiomas is essential because these lesions can cause airway obstruction or narrowing. In adult patients with Sturge-Weber syndrome in whom a difficult airway is anticipated and who can cooperate, awake intubation may be an appropriate airway management strategy, in contrast to pediatric cases.

## Conclusions

We report the successful management of an anticipated difficult airway in a patient with Sturge-Weber syndrome using awake intubation. The decision to perform awake intubation was based on a careful preoperative assessment, particularly airway evaluation using CT. In patients with Sturge-Weber syndrome undergoing general anesthesia in whom difficult mask ventilation is anticipated, anesthesiologists should carefully evaluate preoperative imaging. They may consider awake intubation for anesthesia induction and awake extubation during emergence to enhance patient safety.
